# Enhancing the use of economic evidence in vaccination policy and decision making in low- and middle-income countries: a scoping review of existing strategies

**DOI:** 10.1136/bmjopen-2025-103992

**Published:** 2025-12-28

**Authors:** Chinyere Cecilia Okeke, Onochie Eze, Chinazom N Ekwueme, Oluchukwu Ezechukwu, Chinyere O Mbachu, Benjamin Uzochukwu, Obinna Onwujekwe

**Affiliations:** 1 Department of Community Medicine, University of Nigeria, Nsukka, Nigeria; 2 Health Policy Research Group, University of Nigeria, Nsukka, Nigeria; 3 University of Nigeria, Nsukka, Nigeria

**Keywords:** Vaccination, Decision Making, HEALTH ECONOMICS, Health Services, Health policy

## Abstract

**Abstract:**

**Objectives:**

The use of economic evidence to prioritise vaccines and delivery strategies to optimally use in immunisation systems is becoming a global priority, especially in low- and middle-income countries (LMICs), in view of challenges in funding and the need to make more efficient use of available resources. We undertook a scoping review to identify and synthesise available evidence on strategies that have been used to enhance the use of economic evidence in policy and decision-making in the immunisation ecosystem in LMICs. The review was also used to identify the facilitators and constraints to the use of economic evidence for vaccination policy and decision making in LMICs and the sustainability of the identified strategies.

**Design:**

A scoping literature review was undertaken to generate the evidence. The review adhered to the first five steps of Arksey and O’Malley’s methodological framework (identifying and refining the research questions, identifying relevant articles, selection of studies, data extraction and charting and data synthesis) and Preferred Reporting Items for Systematic Reviews and Meta-Analyses extension for scoping reviews.

**Data sources:**

Full-text articles were searched on PUBMED, HINARI and DOAJ using different combinations of search words as of 16 December 2024

**Eligibility criteria for selecting studies:**

We included articles from LMICs, including Africa, and global experiences, including those from LMICs. Papers must be written in English or have an English language translation available and published between 1 January 2004 and 16 December 2024.

**Data extraction and synthesis:**

Two independent reviewers used standardised methods to search, extract, and screen included studies. The findings from the review were summarized in themes that were synthesized qualitatively.

**Results:**

18 eligible articles met the inclusion criteria and were included in the synthesis. It was found that economic evidence was systematically requested and demonstrably influencing vaccine introduction or prioritisation decisions in only eight out of 32 LMIC settings with functional National Immunization Technical Advisory Groups (NITAGs) and in fewer than 20% of documented new vaccine introduction processes since 2015. In the majority of cases, decisions were reported as being driven primarily by disease burden, political priority, donor recommendations or historical precedent, with economic analyses either absent, produced post hoc or acknowledged but not used as a decisive factor.

**Conclusions:**

There is minimal use of evidence from economics in decision-making within the immunisation ecosystem. Expert advisory committees in LMICS can, however, enhance the use of economic evidence in vaccination policy and decision-making. Hence, in order to use economic evidence for decision making, national advisory committees such as NITAGs need enhanced capacity, independence and close collaboration with researchers. LMIC NITAGs could also benefit from tailored adaptations, such as simplified cost-effectiveness tools and regional economic data hubs, to bridge this gap in decision-making and bring economic evidence to the fore of their decisions.

**Strengths and limitations of the study**

- This review used a broad search strategy to identify a wide range of published evidence and comprehensively synthesised available evidence on strategies that have been used to enhance the use of economic evidence in policy and decision-making in the immunisation ecosystem in low- and middle-income countries (LMICs).
- The review highlights the challenges of the use of economic evidence for improved decision making and how the challenges can be overcome in LMICs.
- There is a wide variety in terms of rigour and quality of the studies we used in our review.
- The study did not use a wide range of bibliographic databases. There is, therefore, the possibility that relevant articles were missing, though the databases searched were the most common sites for LMICs, especially African publications.
- The use of only articles published in English would mean that some relevant information contained in articles published in other languages would be missed.

## Introduction

 The use of economic evidence is essential for ensuring that research findings effectively advise policy and practice, thereby helping to close the gap between evidence and decision-making.^1^ Although policies should be informed by evidence, this is not always the case in most low- and middle-income countries (LMICs).^2^,^4^

As many LMICs such as Nigeria prepare to take over the responsibility for funding their immunisation programme, policymakers and programme managers need to use economic evidence in deciding which vaccines to prioritise and which vaccination strategies to adopt. Such evidence will ensure optimal value for money for their investments and also provide for efficient and equitable use of domestic resources in supporting procurement and delivery of high-priority vaccines.

Translating economic evidence generated from research into policy and practice involves bridging the gap between what is known through research and what is implemented in actuality.^5^ The phenomenon is also related to the wider issue of translating research findings into policy practice, which has remained a big challenge worldwide.

In some cases, evidence from economic evaluation is used in healthcare decision-making if the evidence is accessible and acceptable.^6^ Economic evaluation is defined as the comparative analysis of alternative interventions in terms of both their costs and effects.^7^ Economic analysis is an intrinsic factor in formulating policy decisions and identifying the most efficient vaccination procedure at the country level.^8^ It has the possibility of guiding complex programmes,^9^ supplying answers to questions^10^ and informing resource allocation decisions.^11^

Introducing uptake of vaccine-related economic evidence into a research design and process has contributed to some improvements in Nigeria’s vaccine decision-making, such as the introduction of rotavirus^12 13^ and decisions on COVID-19 vaccination strategies.^14 15^ Furthermore, research funders are now requesting evidence of their research findings in decision-making processes and policymaking,^16 17^ hence the need to highlight its addition in research design.

Factors that limit the translation of findings from economic evaluations and other economic evidence into vaccine policy and decision-making include poor quality of research informing economic evaluations, conflicts of interest, negative attitudes to healthcare rationing, difficulties in transferring resources between sectors, and the absence of equity considerations.^18 19^ There are also delayed communication of research outputs, lack of demand for research, and issues of trust^16 17 19^ and inaccessibility of evidence from economic analysis and evaluations to policymakers, plus the lack of capacity to evaluate the quality of the evidence.^19^

Strategies to overcome these barriers have been suggested in the literature, including capacity building, rapid evaluation, structured abstract databases, reporting checklists, adherence to general guidelines for economic evaluations and considering factors other than cost-effectiveness in economic evaluations, such as equity or budget impact.^18 19^ One emerging tool for National Immunization Technical Advisory Group (NITAG) assessment, the NITAG Maturity Assessment Tool (NMAT), has been piloted in LMICs to evaluate maturity across seven domains, including processes for evidence integration, with initial findings highlighting resource constraints as a key barrier in 91% of countries assessed. This maturity grid is designed not just to measure but to guide progressive strengthening over time. It has since been adopted by WHO, Gavi and several regional networks, such as the African Region (AFR)-NITAG and Asia-Pacific NITAG Network, for capacity-building plans.^20^

In high-income countries (HICs), integration of economic evidence into immunisation decision-making is often formalised and follows a structure, serving as a model for global policy.^21^ For instance, in the UK, the Joint Committee on Vaccination and Immunization routinely requires health economic assessments, including cost-effectiveness analyses aligned with National Institute for Health and Care Excellence methods, to inform recommendations on vaccine inclusion in the National Health Service schedule.^22^ The government is obliged to provide appropriate funding if this is met. This was the case for the introduction of the meningococcal B vaccine following a revised cost-utility analysis.^23^,^25^ Similarly, in the USA, the Advisory Committee on Immunization Practices (ACIP) considers economic evidence such as cost-effectiveness ratios and budget impact analyses, alongside clinical and epidemiological data, with explicit Centers for Disease Control guidance, ensuring transparency and methodological rigour in presentations to the committee.^26 27^ Positive ACIP recommendations activate mandatory insurance coverage under the Affordable Care Act, expanding the role of economics in equitable access.^28^

These processes by the HICs on the use of economic evidence for decision making highlight the potential for economic evidence to drive evidence-based prioritisation. However, the evidence on a similar occurrence in LMICs is unclear >potentially, LMICs may face some context-specific barriers in adapting such best practices in evidence-based decision making (EBDM) and implementation in the immunisation ecosystem.

This review identified and synthesised available evidence on strategies that have been used to enhance the use of economic evidence in policy and decision-making in the immunisation ecosystem in LMICs. It provides the basis for ways and means of enhancing the use of economic evidence for making optimal policies and decisions in priority setting and implementation in the choice of vaccines and their delivery strategies in LMICs. Hence, it contributes to the development of systematic methods to guide how economic evidence should be generated and used in vaccine policy and decision-making.

## Methods

A scoping review of literature was undertaken to identify and synthesise current evidence on strategies that have been used to enhance the use of economic evidence in vaccination policy and decision-making.

The first five steps of Arksey and O’Malley’s methodological framework were adhered to in the scoping review.^29^,^31^ The Preferred Reporting Items for Systematic Reviews and Meta-Analyses (PRISMA extension for scoping reviews was also adopted.^32^

### Search strategy

A literature search was performed in PUBMED, HINARI and DOAJ using specified search strategies. These three databases were chosen following the Arksey and O'Malley framework,^29^ which calls for a repeated literature search. A literature search should map the concepts, the evidence types and the gaps in the field, and it does not try to be complete.

PUBMED is a database that covers a range of peer-reviewed health science literature, thus a good source for studies on immunisation economic evaluations and policymaking. HINARI is part of the WHO’s Research4Life initiative and was selected to prioritise accessibility for researchers in LMICs, as it provides free or low-cost access to a vast array of health-related journals that may otherwise be paid for, ensuring inclusion of contextually relevant evidence from resource-constrained settings. DOAJ was included to capture high-quality open access journals, which are particularly prevalent and important in LMICs, where subscription barriers limit dissemination and uptake of research. This combination of databases balances breadth, relevance and feasibility, especially given the review’s focus on English-language publications from 2004 onward and the supplementation through reference list screening to identify additional articles. Limiting these databases also addressed practical constraints such as time, resources, and avoiding overlapping indexes, while still yielding a representative sample of the literature for thematic synthesis.

The search on the databases was performed on 16 December 2024. These search strategies were developed using a combination of keywords, Boolean operators and relevant truncations. Table 1 shows the search terms that were used for each database. Reference lists (bibliography) of identified literature were reviewed for relevant articles.

**Table 1:** List of databases and search terms used for the literature search

Database	Search terms/strategy	Filters
PUBMED	((((immunization[Title/Abstract]) OR (vaccination[Title/Abstract])) AND (policymaking[Title/Abstract])) OR (decision making[Title/Abstract])) AND (economic evidence[Title/Abstract])	Year filter: 2004–2024 Language filter: English
HINARI	“vaccination policy” OR “immunization policy” AND “decision making” OR “economic evidence”	Year filter: 2004–2024
DOAJ	immunization policy making OR immunization decision making OR vaccination policymaking OR vaccination decision making OR economic evidence Vaccination policy making OR immunization policy making OR immunization decision making OR vaccination decision making	Default year filter: 2004–2024

1) PUBMED, Is a search engine for Biomedical Research by the US National Library of Medicine 2) HINARI meaning Health InterNetwork Access to Research Initiative by World Health Organization. 3) DOAJ meaning Directory of Open Access Journals by Infrastructure Services for Open Access.

### Identifying relevant articles

Articles reporting quantitative and qualitative research methods or mixed methods, programme reports, expert reviews and commentaries written in English or having English language translation available and published between 1 January 2004 and 16 December 2024 were considered eligible for the review. Post-2004 literature captures Gavi era, the rise of NITAGs and increased use of economic evaluations. The geographic scope of the review was articles from Africa and other LMICs. Articles on global experiences, including from LMICs, were included in the review. Protocols, editorials, opinion pieces and articles with unidentified authors were excluded. Articles reporting studies not exclusively conducted in Africa or LMICs were included, provided they were highly relevant and provided rich information.

The search was conducted in English. Each search term was allocated to two independent reviewers and data was extracted using Excel sheet with the following headers: (1) document source; (2) search term; (3) full reference or citation of article; (4) full text or abstract/summary available; (5) country or region; (6) type of article (research, evaluation report, workshop presentations, conference proceedings, academic blog, etc). We applied some eligibility criteria as shown in table 2 below.

**Table 2:** Eligibility criteria used for screening titles/abstracts and full-text articles

Component	Inclusion criteria	Exclusion criteria
Publication date	1 January 2004–16 December 2024	Studies published before 2004 or after 16 December 2024
Language	Published in English or with an available English-language translation or full text	Not available in English and has no English translation/full text
Study focus	Studies that address the use or strategies to enhance the use of economic evidence in immunisation/vaccination policy-making or decision-making processes Studies that show economic evidence explicitly include cost-effectiveness analysis, cost-utility analysis, cost-benefit analysis, budget-impact analysis, health technology assessment, fiscal space analysis or any other form of economic evaluation relevant to vaccine prioritisation, introduction or delivery strategies.	Purely methodological papers on how to conduct economic evaluations without discussing their application or uptake in policy processes Clinical, epidemiological or immunological studies of vaccines with no linkage to policy-making, priority-setting, resource allocation or use of economic evidence
Geographic scope	Studies with a primary focus on LMICs in Africa or elsewhere or Global or multicountry studies that explicitly include and discuss LMIC experiences or contexts,	Focused exclusively on high-income countries with no discussion of relevance or applicability to LMICs.
Document type	Peer-reviewed original research (quantitative, qualitative or mixed-methods), systematic or scoping reviews, programme evaluations/reports, expert commentaries or policy analyses that present empirical findings or documented experiences	Editorials, letters, opinion pieces or commentaries that present no empirical data or documented country/programme experiences Articles with no identified author(s) or institutional affiliation Study protocols, trial registrations or conference abstracts without accompanying full-text results
Relevance	Studies that contain information on specific strategies/mechanisms to promote uptake of economic evidence; role of advisory bodies (eg, NITAGs) in using economic evidence; facilitators or barriers to translating economic evidence into policy/decision-making and documented outcomes or impact of such strategies in LMICs	Duplicates or near-duplicates of already included publications

LMIC, low- and middle-income country; NITAG, National Immunization Technical Advisory Group.

### Selection of studies

Identified studies were screened independently by four researchers (CM, CE, OnE and OiE). In the first phase, the titles and abstracts of the studies were screened using the eligibility criteria in table 2. Articles that did not meet the inclusion criteria were screened out. In the second phase, full texts of articles that passed the initial screening phase were reviewed. Articles that were not relevant to the objectives of the scoping review were further excluded.

Discrepancies between reviewers’ decisions on study selection were resolved through discussion, and no tie-break was needed.

### Data extraction and charting

Full-text extraction was carried out in Excel by three authors (CO, CM and OE) using a data extraction sheet that was developed for the review with the following headings, (1) full citation of article; (2) name and description of approach; (3) stakeholders involved and their roles; (4) type/format of evidence promoted/used (eg, research, administrative data, programme evaluation, etc); (5) facilitators and challenges; (6) sustainability of approach; (7) impact (effects, outcomes of approach. Inter-rater reliability for title and abstract screening was substantial (Cohen’s κ=0.73, 95% CI 0.69 to 0.77; 84% crude agreement) and almost perfect for full-text screening (κ=0.89, 95% CI 0.84 to 0.94; 92% agreement) across the pairs of reviewers. Emerging discrepancies in the data extracted were resolved by consensus.

The assumptions or simplifications made and the justification for making them are as follows:

1. Economic evidence such as cost-effectiveness, budget impact and health technology assessment (HTA outputs: this was focused on standardised forms of economic evaluation relevant to vaccine prioritisation; it excluded macroeconomic analyses unless linked to immunisation decisions.
2. Advisory committees are the primary institutional mechanism: the review centred on NITAGs and similar bodies as the main conduit for economic evidence; other pathways include direct ministry use and parliamentary reviews, but these were not systematically extracted.
3. LMIC experiences are generalisable within similar contexts: assumed common challenges and facilitators across diverse LMICs (eg, Nigeria, India, Indonesia) despite heterogeneity in health systems.
4. Bias for use of only English: our inclusion was limited to English or translated articles; thus, under-represented non-English evidence from francophone African or Asian countries. This has been stated as a limitation of the study.
5. Publication date greater than 2004 reflects the modern evidence era: we assumed post-2004 literature captures the GAVI era, the rise of NITAGs and the increased use of economic evaluations, so pre-2004 evidence was deemed less relevant to current systems.
6. There was no meta-analysis as we assumed qualitative synthesis was sufficient: most of the studies had heterogeneous study designs and outcomes, including quantitative pooling; hence, thematic synthesis was deemed appropriate for our scoping review objectives.
7. Two-reviewer screening was seen as sufficient rigour: we assumed independent dual review with consensus resolution minimised selection bias; no third reviewer or kappa statistics were reported.
8. Global experiences, including LMICs, were seen as a valid proxy: we included global reviews or HIC models only if they explicitly discussed LMIC adaptation or inclusion.

### Critical appraisal of individual sources of evidence

#### General appraisal framework

A single tool (Excel sheet) was used for the extraction, but for reviews or commentaries, we used the authority, relevance and conflict of interest assessment.

#### Independent dual rating

Two reviewers independently assessed each study using a standardised extraction form with yes/no/partial/unclear ratings per criterion, and discrepancies were resolved via consensus or a third reviewer.

#### Reporting of appraisal outcomes

We used a narrative, indicating the proportion of studies meeting each criterion. There were common weaknesses, such as a lack of local data justification and undefined success.

#### Sensitivity consideration

A subgroup narrative synthesis was conducted to explore whether excluding low-quality studies alters key themes. For example, we checked if NITAG effectiveness holds only in high-quality reports.

### Synthesis of results

Data were analysed using thematic synthesis following the approach described by Kavanagh *et al*^33^ and adapted for scoping reviews.^34^ The process was iterative and combined both deductive and inductive elements.

#### Thematic development

The initial framework was prespecified. An initial coding framework was developed deductively from the review objectives and the data-extraction template. Themes included: types of strategies/mechanisms; role of advisory committees (NITAGs, regional, global); facilitators of uptake; barriers/challenges; reported outcomes and sustainability. The reviewers independently coded the ‘results/findings’ and ‘discussion/conclusions’ sections of all included studies line-by-line. New codes that did not fit the initial framework were created inductively, for example, late engagement of health economists, use of simplified one-page policy briefs, political championship, donor-driven versus country-owned processes. There was then the development of descriptive themes, with codes grouped into descriptive subthemes through constant comparison and regular team discussions. For example, the prespecified theme ‘facilitators’ expanded into the final subthemes: capacity building/training, user-friendly evidence formats, early stakeholder engagement, local data generation and sustainable domestic funding. Then, analytical themes were generated from the descriptive themes that were interpreted to generate higher-order analytical themes: ‘institutionalisation of economic evidence that requires both technical capacity and political legitimacy.’

#### Weighting of evidence and confidence assessment

During synthesis, findings reported in multiple sources or supported by higher-quality empirical studies were given greater prominence. Single-source or purely assertive statements from low-rigour reports were retained for completeness but clearly stated as ‘suggestive’ in the results, for example, NITAGs in well-documented African settings were presented with greater confidence than those from single, weak reports.

#### Barrier/facilitator validation

Challenges were only included if substantiated beyond assertion.

#### Discussion of limitations

This has an explicit section in this manuscript.

#### Recommendations

We prioritised actions supported by robust evidence, for example, investing in training was backed by a training programme evaluation.

This was iteratively refined until the review team agreed that it comprehensively represented the dataset. The final themes and subthemes are presented in the Results section. The synthesis included useful information on the underlying processes, effects/outcomes and facilitators or challenges to promoting the use of evidence (including economic evidence) in vaccination policy and decision-making.

## Findings

**Figure 1:**
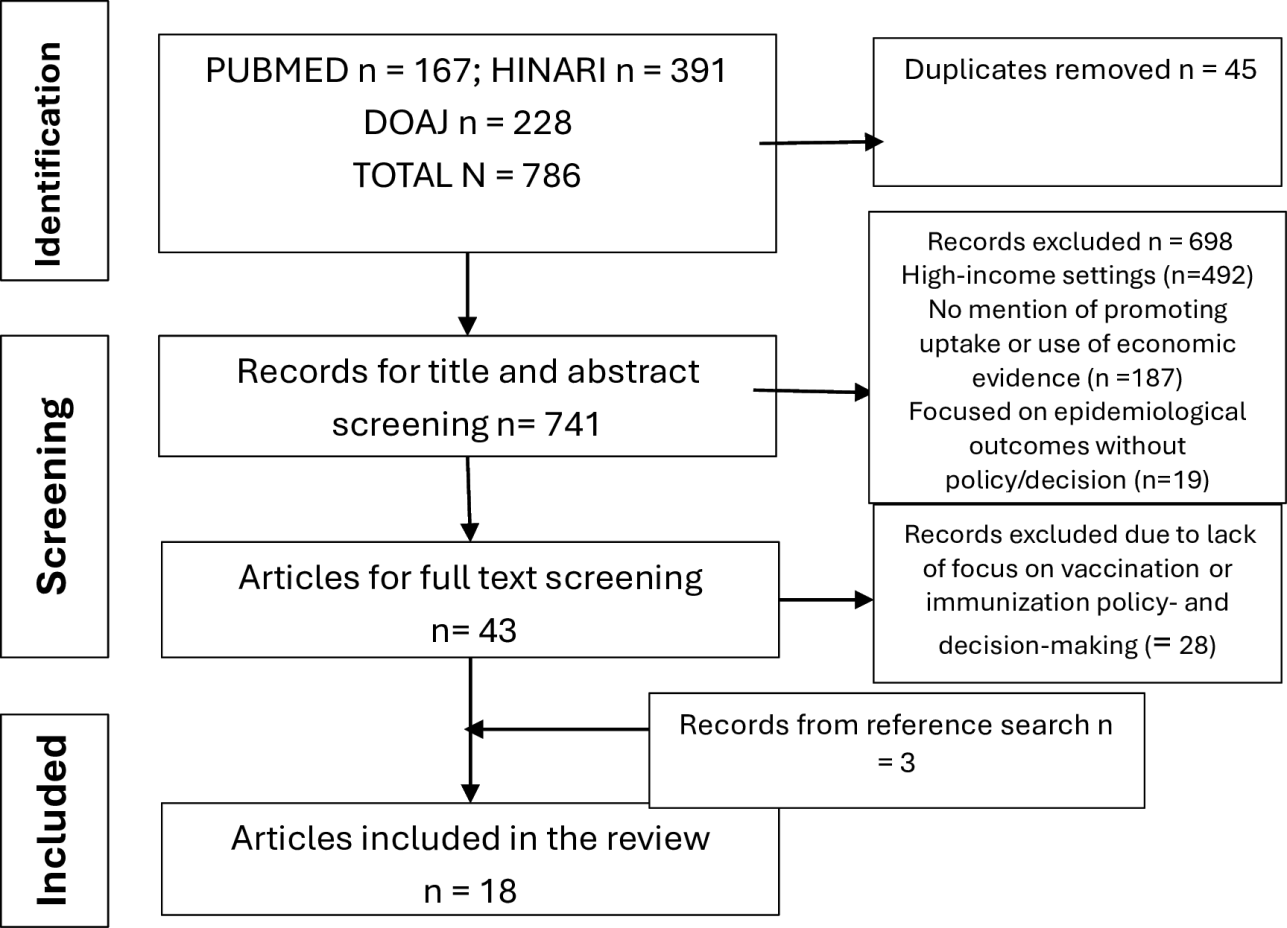
Preferred Reporting Items for Systematic Reviews and Meta-Analyses flow diagram of the stages of the scoping review.

Figure 1 shows the PRISMA flow diagram of the different stages in the scoping review. A total of 786 articles were found using the search terms outlined in table 1. A total of 45 duplicates were removed. The titles and abstracts of 741 articles were screened using the eligibility criteria and 698 were removed. Full texts of 43 articles were screened, and their reference lists were searched for relevant articles. Articles that did not have a focus on vaccination or immunisation policy-making and decision-making were excluded at this stage. A total of 18 articles were included in the final review, including three that were identified from a reference search (figure 1).

The articles included in the review are summarised in online supplemental table S1. The review identified two broad groups of strategies that have been used to enhance the use of economic evidence in vaccination policy and decision-making, and they were: (1) the use of expert advisory committees and (2) the use of processes and frameworks to align economic evidence to vaccine policy priorities and to track progress, demonstrate accountability and solicit funding for immunisation.

14 articles reported the use of expert advisory committees to promote evidence-based vaccination policy and programme decision-making in LMICs. Three of these articles reported the use of a global expert advisory committee on vaccination, the WHO Strategic Advisory Group of Experts (SAGE),^35^,^37^ while 11 articles reported the use of national expert advisory committees on immunisation, including the NITAGs and^37^,^44^ Zambia’s Inter-agency Coordinating Committee and the Pasteur Institutes around the world.^45^

14 articles reported the use of expert advisory committees to promote evidence-based vaccination policy and programme decision-making in LMICs. Three of these articles reported the use of a global expert advisory committee on vaccination, the WHO Strategic Advisory Group of Experts (SAGE),^35^,^37^ while 11 articles reported the use of national expert advisory committees on immunisation, including the National Immunization Technical Advisory Groups (NITAGs) and^37^,^44^ Zambia’s Inter-agency Coordinating Committee (ICC)^47^ (and the Pasteur Institutes around the world.^45^

Four articles identified the use of processes and frameworks to align vaccine economic evaluations with policy priorities^19 36 37^ and to use economic evidence to track progress, demonstrate accountability and solicit funding for immunisation.^11^ HTA was used in Nigeria as a structured evaluation process to support EBDM for COVID-19 vaccination.^37^ Full Value of Vaccine Assessments (FVVA) framework is a multidimensional evaluation approach that was used to align vaccine value assessments with health policy priorities.^36^ Accessibility and acceptability frameworks were used to review the barriers to using evidence from economic evaluation in healthcare policymaking.^19^ One article explored how Beyer and Trice’s organisation’s science conceptual framework is used to present epidemiological and economic evidence for tracking progress, demonstrating accountability and soliciting funds for immunisation activities. It uses a conceptual framework and interviews with mathematical modellers and employees of global health organisations. The findings provide insights into research utilisation in organisations and evidence-based management.^11^ A list of the 18 included articles is presented in online supplemental table S1.

### Processes of influencing vaccination policy and decision-making

The processes of influencing vaccination policy and decision-making are described separately for each approach.

#### Expert advisory committees

SAGE is an independent advisory group to the WHO on vaccine policy and strategy.^35^,^37^ The group comprises renowned immunisation, vaccine and public health experts from a broad range of disciplines and professional affiliations, and its scope of work encompasses all vaccine-preventable diseases.^35 36^ SAGE’s processes of influencing vaccination policy and decision-making begin with a request from WHO for recommendations, followed by the development and communication of recommendations.

The development of recommendations by SAGE begins with the review of published and unpublished evidence, followed by the development of options for recommendations by a subcommittee or working group comprising 2–3 SAGE members and additional experts. Where necessary, research can be commissioned to fill the evidence gap.^36 37^

Recommendations take into consideration epidemiologic, clinical, vaccine and immunisation, economic contexts and coordination with other preventive interventions, for example, malaria vaccination as part of malaria control/elimination programme. Quality of evidence is assessed using the Grading of Recommendations Assessment, Development and Evaluation approach.^37^

Communication of recommendations from SAGE is through position papers—a summary of vaccine and vaccination information on large-scale vaccination programmes.^37^ Position papers contain information on specific disease epidemiology and available vaccines (including economic evidence on cost-effectiveness). Target audiences are government policy/decision makers and immunisation programme managers, funders, industry, professionals and academia/research community. One-page summaries and PowerPoint presentations from position papers are prepared and posted on the SAGE website, alongside meeting presentations and background documents that inform recommendations.^36 37^

NITAGs comprise a range of experts who primarily advise Ministry of Health (MOH) on new vaccines, including technical and cost-effectiveness issues.^40^ They are recognised as important partners and contributors to immunisation policy.^44^ NITAGs are particularly valued for providing an independent EBDM process that is nationally owned.^41^ Their main role is to promote evidence-based vaccination policy and programme decision-making^46^ and guide country-level adaptation of global and regional recommendations, such as adjusting existing programmes and schedules.^45^,^47^ More powerful NITAGs are involved in decision-making on revising national immunisation schedules and approving funding.^42^

The process of influencing vaccination policy and decision-making, as reported by NITAGs, often begins with a request from the MOH for recommendations.^38 44^ The rest of the process then involves question formulation, evidence gathering, quality assessment and analysis, and consensus recommendation.^38^

NITAGs have reported using published and unpublished evidence from research and programme data, commonly epidemiologic and economic evaluation data.^40 42^ WHO recommendations are also commonly used.^42^ Other types of data that are less commonly used by NITAGs are data on affordability and financial sustainability, vaccine availability and supply, clinical characteristics or vaccine effectiveness and data on social and equity considerations.^42^ Although it is unclear from the literature how data on social and equity considerations have been used to inform recommendations of NITAG.

A review of NITAG’s roles in Indonesia on the polio eradication end game reported that following a request for recommendations from MOH on the introduction of inactivated polio vaccine, a disease-specific task force was set up to assess and identify facilitators and constraints to introducing a new vaccine. The task force presented results of their assessment to plenary (the rest of the members of NITAG), and NITAG piloted the vaccine introduction in the country’s immunisation programme to generate evidence of outcomes (predefined elements) that informed recommendations to the MOH.^42^ Morocco’s NITAG leveraged standardised assessment tools and immunisation programme records.^46^

A review of the role of Pasteur Institutes in vaccination decision-making shows that they are a non-profit organisation, with core and non-core members who synthesise data on the burden of disease from their laboratory surveillance activities and provide specific information regarding immunology and safety issues related to vaccines, as well as information regarding emerging diseases which they present as reports and PowerPoint presentations to help NITAG in making recommendations to the government.^45^ These are what they have been doing in some LMICs such as Côte d’Ivoire and Viet Nam.

#### Use of processes and frameworks

For articles that reported influencing vaccine policy and decision-making by aligning economic evaluation studies to vaccine policy priorities, evidence was synthesised from national policy documents, stakeholder interviews, health system assessments and global guidelines to produce useful recommendations to policy and decision makers.^48^ Nigeria used HTA reports and stakeholder mapping.^48^ Zambia relied on vaccine coverage data and policy documents.^34^

It was found that the FVVA framework drew on WHO guidelines and global vaccine impact studies.^49^ It has three key elements. First, to enhance assessment by adapting operational value-assessment methods and tools to involve broader benefits of vaccines as well as opportunity costs borne by stakeholders. Second, by using a purposeful process to recognise the stakeholders and ensure country ownership of decision-making and priority setting. Third, the FVVA framework provides a consistent and evidence-based approach that expedites communication about the full value of vaccines, helping to enhance alignment and coordination across diverse stakeholders. FVVA used a mixed methods approach with literature reviews, stakeholder interviews and surveys and quantitative data analyses to assess the vaccine needs, estimate the costs and benefits of delivering the vaccines, as well as identify outstanding areas of need where stakeholder intervention can be helpful. These were communicated to decision-makers in the form of reports.^49^

The accessibility and acceptability framework was used to investigate interventions to improve the translation of evidence from economic evaluation into policy, using capacity-building workshops, standardised formats of presenting results, rapid evaluation techniques, multicriteria decision analysis and awareness-raising initiatives.^19^ It identified a way to evaluate proposed strategies to encourage the use of economic evaluations in policy and decision-making. Interventions that warrant investigation by identifying barriers to accessibility of economic evaluations, including the cost and time required to conduct economic evaluations, difficulties translating economic evaluations into different contexts, decision makers’ lack of time to assess research, poor awareness of current evaluations, poor communication from health economists, the complex nature of economic evaluation design, excessive variation in economic evaluation methodologies and presentation and lack of economic evaluation expertise among decision makers. Also, a need to look at the scientific, institutional and ethical acceptability of the economic evidence produced, as these might determine the ability of decision-makers to use the economic evidence produced.^19^

A review of the use of conceptual frameworks to present economic evaluations to track progress, demonstrate accountability and solicit funding for immunisation showed that the authors made use of literature reviews and conducted indepth and semistructured interviews using Beyer and Trice’s organisation science conceptual framework that addresses possible types of use of evidence throughout different organisational processes with a focus on action generation and knowledge utilisation. It was found that economic evidence is slowly integrated into organisational processes and that it is one of many influences on global health organisations’ actions. The process is guided by relationships and trust developed over time. Economic evidence was used once it was ingrained in the decision-makers’ understanding and the organisation’s agenda. Evidence consumers agree that research evidence is often used to track progress, demonstrate accountability, inform and communicate targets to donors, and support an argument when wanting to receive more funding for immunisation. Continuously engaging stakeholders throughout the research production process, including the setup of the model structure and defining the outcomes that are required, is necessary to maximise the value of the economic evidence produced.^11^

### Effectiveness of identified strategies in influencing evidence-based vaccination policy and decision-making

Findings from the review show that the strategies have had various effects on evidence use for vaccination policy and decision-making. The use of expert advisory committees has had the most documented effects on vaccination policy and decision-making, and it appears to be an effective approach for improving the use of evidence by policy and decision-makers.

Advisory committees have contributed to circumventing the challenges of accessing evidence for policymakers as seen in this excerpt:

In the past, important evidence sometimes did not reach key policymakers in a timely and effective manner. For example, policymakers indicate consistently that their decisions require data for the burden of vaccine-preventable disease, the vaccine’s efficacy and safety, and the cost-effectiveness of vaccination, but rarely make the investments needed to generate these data. In the past, even when these data were published in peer-reviewed journals, they did not reach policymakers.^35^

Policy and strategy recommendations from SAGE have formed the basis for all of WHO’s vaccine position papers.^35^ These recommendations have also contributed to (1) adjustment of country immunisation strategies; (2) successful introduction of new vaccines into country immunisation programmes—rotavirus and pneumococcal conjugate vaccines; (3) development of integrated strategies for disease control, for example, coadministration of intermittent preventive therapy for malaria during immunisation visits and (4) adoption of innovative financing mechanisms for vaccines in LMICs.^36^

The effectiveness of NITAGs varies depending on the context, NITAG maturity and support received from government and development partners.^42^ Some influential global actors are not convinced of the value of NITAGs in facilitating or derailing vaccine introduction timelines in LMICs. Hence, the lack of support—global funding—of NITAGs. However, some LMIC NITAGs have substantially influenced evidence-based vaccination policy and decision-making in their countries.^40 44^ Reports show that evidence-based recommendations from NITAGs have informed vaccine decision-making^8 40 44^ and introduced new and underused vaccines into the national schedule.^8^

For instance, the Ugandan NITAG’s recommendations informed the country’s Immunization Bill (now a law); the introduction of new vaccines into the country’s immunisation programme and the adoption of interventions to improve coverage of existing vaccines within the country.^42^

NITAGs’ credibility and preference for local evidence also improved country ownership of vaccine policy and decision-making.^41 44^ It was reported that NITAGs valued local data, and members leveraged their networks and affiliations to obtain otherwise inaccessible data and documents.^40^ Consulting local data and context in making recommendations was identified as a key value of NITAGs to policy and decision-making. In Zambia, coordinated efforts improved vaccine coverage.^34^

Nigeria’s HTA process enhanced EBDM for COVID-19 vaccination.^48^ The FVVA framework aligned vaccine policies with health priorities, improving equity and accessibility.^49^ Morocco’s NITAG strengthened policy integration and advisory capabilities, contributing to more effective immunisation strategies.^12^

Economic evidence from mathematical models of vaccine-preventable diseases was found effective for strategic thinking, planning purposes, accountability and advocacy purposes when soliciting funding from external partners for immunisation purposes.^11^ It is also used when supporting evidence is needed to make a case for investing in a particular project over another one.^11^ However, having the evidence users set the economic research agenda did not lead to evidence use, as findings did not lead to vaccine resource allocation decisions as planned.^11^

### Facilitators and challenges to strategies for enhancing the use of evidence in vaccination policy and decision-making

Table 3 summarises the facilitators and constraints of strategies for enhancing the use of evidence in vaccination and decision-making. Notable facilitators include the availability of technical expertise within advisory committees and the availability of standardised tools for economic evaluation.^19 42^ Capacity in health economics was frequently mentioned as a missing expertise among many NITAGs.^42^ Other notable constraints include the limited availability of evidence on the costs and benefits of vaccination and the absence of multicriteria decision analysis for making explicit findings when more than one economic evaluation is done.^35^ A summary of the key reported facilitators and barriers specific to the use of economic evidence in vaccine decisions is presented in table 4.

**Table 3:** List and definition of all variables for which data were sought and any assumptions and simplifications made.

Variable	Definition	How it was operationalised in the review
Strategies to enhance the use of economic evidence	Specific mechanisms, frameworks or interventions used to integrate economic analyses (eg, cost-effectiveness, budget impact) into immunisation policy and decision-making.	They were coded as primary outcome (eg, national expert advisory committees, health technology assessment).
Type of advisory committee	Organisational structure providing technical advice on vaccine introduction/prioritisation using economic evidence.	This was subcategorised as: national (eg, NITAGs); regional; global, which has LMIC representation.
Effectiveness of advisory committees	The degree to which committees successfully influenced policy using economic evidence.	We conducted a qualitative assessment based on reported outcomes; this varied by context and support received.
Contextual factors affecting effectiveness	External or structural conditions shaping committee performance	Included: government support, development partner funding, political environment and the level of the institution.
Missing expertise in health economics	Absence of technical capacity to interpret or generate economic evidence within advisory bodies	The frequently reported gap was extracted as a barrier.
Facilitating factors	Enablers that improve uptake of economic evidence in decision-making	This was coded thematically: capacity-building/training; access to local data and literature; cross-learning from established committees; strong policy linkages and networks; stakeholder engagement in research and user-friendly evidence presentation.
Challenges/barriers	Obstacles limiting the use of economic evidence	This was coded thematically: limited access to local data; lack of transparency; political interference; unsustainable funding; role ambiguity; absence of a standardised evidence format; late involvement of decision-makers.
Use of health technology assessment	Formal processes to evaluate vaccines using economic and clinical evidence	This was extracted as a distinct strategy.
Level of economic evidence use	Extent to which economic analyses inform immunisation decisions	Qualitatively summarised as low to moderate across LMICs
Recommendations for improvement	Specific actions to strengthen evidence-to-policy translation	These included: sustainable funding; enhanced capacity in health economics; independence of committees; closer researcher and policymaker collaboration

LMIC, low- and middle-income country; NITAG, National Immunization Technical Advisory Group.

**Table 4:** Summary of key reported facilitators and barriers specific to the use of economic evidence in vaccine decisions

Characteristics	Facilitators	Constraints
Institutional demand	Legal mandate requiring economic evidence for new vaccine introduction^49^	Decisions driven by donor priority. Absence of a legal mandate for evidence-based decision-making
Composition and support of NITAGs and	Inclusion of more trained health economists, a dedicated secretariat and a budget^38^ Early involvement of policymakers ensures timeliness of evidence-based recommendations^38^ Availability of regional learning networks^38 42^ Culture of evidence use and communication^35 36^	Lack of health economists^40 42^ Political interference or lack of autonomy of NITAG members^35 42^ Weak country-level capacity to implement and sustain NITAG^41^ Poor linkage with the Ministry of Finance and siloed funding streams^41^ Decision-making processes do not follow a defined set of criteria.^44^ Limited economic evidence (eg, costs and benefits of vaccination)^35^
Capacity building and training	Reinforcement of technical capacity of NITAG^38 42^ Country and regional capacity building programmes on economic evaluation ^38 42^ South-South learning and peer programmes^42^	Limited pool of local health economists^44^ High turnover of trained staff^44^ Lack of sustainability of capacity building programmes^38 42^ Reliance on external consultants; no career track for health economists
Generation and presentation of evidence	Use of locally generated/co-produced epidemiologic and economic evidence.^42^ User-friendly policy briefs and dashboards.^38^ Timeliness of economic evidence for budgetary decision making.^38^	Over-reliance on global or modelled estimates, which are donor-driven or based on unrealistic assumptions^42^ Evidence presented too late in the decision cycle^42^
Stakeholder engagement and political support	Early involvement of Ministry of Finance and the parliament Strong political championship Multi-stakeholder platforms Co-production of evidence with policymakers^48^ Long-standing relationships	Donor-driven agendas dominating decisions Weak demand for evidence from decision-makers Mistrust between researchers and policymakers
Institutional and financial sustainability	Domestic budget lines for NITAGs/HTA units^42^ Region al resource pooling	Donor withdrawal/Gavi transition^42^ Fiscal constraints due to competing health priorities
Aligning economic evaluation studies to vaccine policy priorities using frameworks	Availability of standardised evaluation tools^19^ Research agenda setting with clear route to evidence use^11^ Evidence relevant to the agenda and understood by decision makers^11^	Lack of standardised formats for results Lack of multicriteria decision analysis^19^ Late or lack of continuity of involvement of evidence consumers in evidence generation^11^

HTA, health technology assessment; NITAG, National Immunization Technical Advisory Group.

### Sustainability of identified strategies

Sustainability of expert advisory committees such as NITAG depends on its institutionalisation,^50^ availability of dedicated budgets and staff, technical support from partners and perceived value by the global community.^41 44^ Sustained collaboration with global partners also enhanced the longevity of these expert committees,^43^ vaccine introduction and scale-up.^49^ Several NITAGs that relied on external support are facing sustainability challenges.^40^ To ensure structural and functional sustainability, institutional integration is needed, as it also enhances the acceptance of expert review processes.^8^

The sustainability of the use of frameworks depended on integrating evaluation frameworks into national policies, continuous capacity building of stakeholders to interpret the economic evidence produced and securing long-term funding.^19 48 50^ Also, on the relationship of the evidence producers with policymakers and evidence users, as well as the availability of institutional structures for the uptake of research evidence.^11^

## Discussion

This scoping review shows that the systematic use of economic evidence in immunisation decision-making remains inconsistent across LMICs. The strategies identified in this review operate as institutional and procedural strategies that create formal demand for economic evidence, for example, strengthening NITAGs, legislative mandates, using decision frameworks and knowledge-translation strategies that ensure locally produced economic analyses are perceived as timely, relevant, credible and actionable by decision makers. Both levels are required for economic evidence to move from availability to actual influence on vaccine introduction, financing and delivery decisions.

The review identified national, regional and global expert advisory committees as effective strategies for enhancing the use of economic evidence in vaccination policy and decision-making by giving independent expert recommendations based on sound scientific evidence. Their influence is often limited by weak technical capacity, late engagement, donor-driven priorities and political override. They are especially needed for the introduction of new vaccines such as malaria, Ebola, COVID-19, and typhoid fever vaccines,^21 51^ considering limited resources with Gavi withdrawal. The transition from donor to domestic financing represents both a risk and an opportunity. The risk is that the abrupt loss of external technical support can paralyse growing HTA processes. The opportunity is that fiscal pressure forces ministries of finance to demand value-for-money justification, creating new demand for economic evidence if the supply from local capacity and user-friendly products is already in place.

Instituting advisory committees at various levels has been mandated by the Global Vaccine Action Plan and has been useful in vaccine decision-making^2140 52^,^54^ as members of these committees have diverse expertise and they draw on their wealth of knowledge to make recommendations that inform vaccine decisions.^21^ The presence of a functional, independent NITAG with a clear legal mandate and domestic funding is the single strongest predictor of economic evidence being requested and considered.^20^ Countries that established NITAGs early and included health economists from the outset, like South Africa, Morocco and Thailand, consistently performed better than others. Recent tools like the NMAT enable LMICs to systematically diagnose maturity gaps during stakeholder engagement for economic analyses. The presence of a functional, independent NITAG with a clear legal mandate and domestic funding is the single strongest predictor of economic evidence being requested and considered.^20^ Countries that established NITAGs early and included health economists from the outset, like South Africa, Morocco and Thailand, consistently performed better than others. Recent tools like the NMAT enable LMICs to systematically diagnose maturity gaps during stakeholder engagement for economic analyses and prioritise interventions, such as embedding health economists, before Gavi transitions. This aligns with our finding that early-established NITAGs perform better and contrast with HIC models to underscore ambitious adaptations for LMICs. Therefore, LMICs preparing for Gavi transition should prioritise legislative backing and sustained budget lines for NITAG secretariats before 2030.^20^ Therefore, LMICs preparing for Gavi transition should prioritise legislative backing and sustained budget lines for NITAG secretariats before 2030.^20^

However, a frequently mentioned area of missing expertise for national advisory committees is health economics, considering that they need to demonstrate their value to vaccine decision-makers through trade-offs and opportunity cost arguments. For these committees to be responsive, there is a need to build the capacity of advisory committee members in health economics^40^ and engage such specialists to enable the conduct of economic evaluations, as well as interpret findings from economic evaluations. The critical shortage is not general ‘training’ but specifically health economists and modellers able to produce or critically appraise budget-impact and fiscal-space analyses. One-off workshops have limited effects; as such, sustained impact requires in-country postgraduate programmes, regional centres of excellence, for example, following the ProVac model and formal career tracks within ministries of health. Government health institutions are encouraged to engage NITAG and health economists until they are comfortable with demanding and making use of available economic evidence for vaccine decision-making.

The value in the use of frameworks and HTA for economic evaluations to inform global vaccine decision-making has been shown by other studies.^55^,^57^ More so, an identified constraint to the use of economic evidence for decision-making was the lack of a defined set of criteria for decision-making and the lack of a clear evidence-based methodology to gather and evaluate the evidence.^44^ Many frameworks have been identified and used in developed countries to measure and value economic evaluations, such as the use of the value of vaccines framework^55 58^ and measures of broader economic indicators of vaccine impact in informing investment decisions about vaccines,^58^ but specialised skills are needed to conduct these economic evaluations. Many such skills are lacking in LMIC, hence the inability of policymakers to interpret and use economic evidence for decision-making.

Adequate engagement of stakeholders in the early stages of the design and implementation of economic evidence production, as well as long-term collaborations, should be encouraged between the producers and users of vaccine economic evidence. The government should encourage a demand-driven approach to economic evidence production, as studies have shown that it tailors research to context-specific issues that are pertinent to the decision-makers, besides strengthening organisational and individual capacities.^16 59^ However, our findings showed that evidence consumers setting the agenda did not improve the uptake of vaccine impact estimates (VIE) evidence produced.^11^ This could be due to the low level of culture in using evidence in decision-making in LMICs, especially as it was found not to be an explicit requirement for policy and decision-making.^16 60^ However, the findings were used to argue for more investment in health through vaccination.

Policy briefs, one-page budget-impact summaries and deliberative dialogues led by trusted national institutions were the dissemination formats most frequently seen to increase the influence of cost-effectiveness and budget-impact findings on NITAG recommendations and parliamentary budget approvals, as seen in other studies.^17 61^

To ensure research evidence informs policy and practice effectively, some actions are necessary, such as aggregating and summarising research findings clearly and concisely^17^ and highlighting the implications for policy and practice.^62^ Also, by developing targeted knowledge translation strategies that align with the needs and preferences of different stakeholders and using various communication channels to disseminate research findings.^63^ Policies are then formed by translating research evidence into practical recommendations that are feasible and contextually relevant.

There is a need for regular review of uptake of vaccine-related economic evidence strategies to enable the implementation of identified context-specific interventions.^64 65^ A key insight from the reviewed evidence is that no single strategy works uniformly across LMICs; effectiveness is heavily affected by three contextual factors that future implementation efforts must assess and address. First is political stability and governance culture. In places with politically unstable settings, even well-resourced NITAGs were sidelined or overruled. Conversely, in countries with stable multi-party systems and a tradition of technical advisory bodies, eg, South Africa, Morocco, Thailand, the same NITAG model translated into sustained influence on budget allocations. A political championship from legislators emerged as a strong enabler; without it, technical recommendations rarely survived budget negotiations.

Second is the funding and transition phase, and how it is affected by Gavi. During full Gavi support, economic evidence was often produced to meet donor requirements rather than to inform local decisions, which is not an integrated process. In contrast, countries that are already self-financing, for example, Indonesia, showed markedly higher demand from ministries of finance for budget-impact and fiscal-space analyses. Sudden donor withdrawal without prior local capacity building can lead to the collapse of growing HTA units and a shift in politically driven choices.

Third is the existing local research capacity. Where health institutions already had health-economics departments, for example, South Africa and Kenya, the coproduction of evidence and rapid uptake was feasible. In settings with virtually no trained health economists, external consultants dominated, resulting in low ownership, limited transparency of study findings and a lack of trust in the results. Regional initiatives such as the African Collaborating Centre for HTA only succeeded when a minimum of two to three locally trained professionals were already in place to adapt and defend the analyses. Therefore, before selecting or scaling any uptake strategy, decision-makers and technical partners should conduct a rapid contextual analysis covering these three dimensions. Strategies that ignore unfavourable political or capacity contexts are not likely to succeed. There is a need for basic domestic capacity and stable political environments before investing in advanced HTA tools or adopting sophisticated products that can support decision-making.

### Strengths and limitations of the study

This review used a broad search strategy to identify a wide range of published evidence and comprehensively synthesised available evidence on strategies that have been used to enhance the use of economic evidence in policy and decision-making in the immunisation ecosystem in LMICs. While we tried to use a comprehensive search strategy to identify the articles included in the review, we did not use a wide range of bibliographic databases. Also, no separate systematic grey literature search was performed. There is, therefore, the possibility that relevant articles were missing. The primary aim of this scoping review was to map strategies reported in the published and programmatically cited literature rather than to achieve exhaustive capture of all unpublished material. However, the databases searched were the most common sites for African publications. Also, the use of only articles published in English would mean that some relevant information contained in articles published in other languages was missed, especially from the African Francophone countries and Asia. Additionally, there is a wide variety in terms of rigour and quality of the studies we used in our review, and this diversity was not specifically considered when making conclusions.

The quality grading of studies was not used as a criterion for selection. Hence, the included studies were not assessed for quality. Although a general appraisal framework was applied during data extraction, low-quality or purely assertive reports contributed minimally to the final thematic synthesis. This decision aligns with common practice in scoping reviews, where the primary objective is to map the extent, range and nature of evidence rather than to provide a definitive synthesis suitable for strong policy recommendations.^66^,^68^ Nevertheless, the lack of formal quality assessment has implications for the interpretation of the findings. Themes such as the effectiveness of NITAGs, the value of capacity-building initiatives or the reported barriers to economic evidence or uptake rely heavily on programme reports, expert commentaries and observational studies that may be affected by publication bias, selective reporting or vested interests by development partners. Consequently, while the review reliably identifies the strategies that have been attempted or advocated in LMICs, the strength of evidence supporting their effectiveness or generalisability is not conclusive. Policymakers and research funders are advised to interpret the reported successes and facilitators with caution and to prioritise strategies that have been evaluated in higher-quality comparative or longitudinal studies when such evidence becomes available. Future work in this area would benefit from systematic reviews with rigorous quality appraisal to generate more robust conclusions about which interventions most reliably enhance the use of economic evidence in immunisation decision-making. While our scoping review maps strategies, tools like NMAT could rigorously assess their impact on economic evidence uptake, warranting systematic evaluations in post-Gavi LMICs.^20^ While our scoping review maps strategies, tools like NMAT could rigorously assess their impact on economic evidence uptake, warranting systematic evaluations in post-Gavi LMICs.^20^

## Conclusion

There is a variety of strategies used to enhance vaccine decision-making, including the use of expert advisory committees, Health Technology Assessment and frameworks, as well as the use of economic evidence to track progress, demonstrate accountability and solicit funding for immunisation. Enhancing the use of economic evidence in LMIC immunisation programmes does not primarily require new frameworks or more studies. It requires targeted investments in independent advisory institutions with secure domestic funding, a group of locally trained health economists, and early, policy-oriented evidence packaging. While the feasibility of strategies largely depends on the adoption of the enabling strategies identified, sustainability remains a challenge and was seen to be more in systems that institutionalised the identified strategies and continuously engaged the stakeholders. Donors, governments and technical partners should shift resources accordingly to convert the current one-off use into routine, evidence-informed priority-setting. Resource requirements emphasised the need for financial support, capacity building and improved local data collection to inform vaccine decision-making. To expand the reach and impact of the use of economic evaluations, future initiatives should focus on flexible, scalable strategies, adaptable to various policy contexts, to further improve vaccine policy impact, especially in Africa and other LMICs.

## Supplementary material

10.1136/bmjopen-2025-103992 online supplemental table 1

## Data Availability

Data are available upon reasonable request.
