# Supplementary material for: Enhancing the use of economic evidence in vaccination policy and decision making in low- and middle-income countries: a scoping review of existing strategies
Source: BMJ Open. 2025 Dec 28;15(12):e103992. doi: 10.1136/bmjopen-2025-103992 (PMC12750769; doi:10.1136/bmjopen-2025-103992)
Supplement: online supplemental table 1 [file bmjopen-15-12-s001.docx]

**Table S1:** Summary of articles included in the scoping review

| **Authors** | **Approach described/ evaluated** | **Study aims & methods** | **Country/ region** |
| --- | --- | --- | --- |
| Howard et al [41] | Expert advisory committee | Aim: To examine NITAGs' value, sustainability, and need for support in low and middle-income countries  Method: A mixed-methods study including 130 global and national-level key informant interviews. | LMICs |
| Howard et al [40] | Expert advisory committee | Aim: To examine functionality, quality of recommendation development, and integration of NITAGs with national decision-making bodies and processes.  Method: Comparative case study of six LMICs. Qualitative research method. | Armenia, Ghana, Indonesia, Nigeria, Senegal and Uganda. |
| Levine et al [35] | Expert advisory committee | Aim: Review of challenges to the introduction of new vaccines in developing countries  Method: Literature review of country experiences | LMICs |
| Harmon et al [39] | Expert advisory committee | Aim: Mixed-method study to develop a framework for evaluating governance of NITAGs.  Method: Comprises online survey of national representatives of 40 Global NITAG Network (GNN) country members; Review of legal and policy instruments of 28 GNN member countries; and Case study of Cote D'Ivoire's NITAG | LMIC –Cote d’Ivoire |
| Ba-Nguz et al [38] | Expert advisory committee | Aim: a reflection of the authors’ experience on NITAG’s role in Indonesia (ITAG) and Uganda (UNITAG) on the polio eradication Endgame plan.  Method: Reflections of participants | Indonesia  Uganda |
| Duclos et al [36] | Expert advisory committee | Aim: Special Report on the process and impact of development of immunization policy recommendations at the global level  Method: Cross sectional study that describes the process of development of immunization policy recommendations at the global level and some of their impacts | Global experience including from LMICs |
| Duclos et al [37] | Expert advisory committee | Aim: Describes the process of development of immunization policy recommendations at the global level and some of their impacts  Method: Literature review | Global experience including from LMICs |
| Bell et al [42] | Expert advisory committee | Aim: Assessing the remit, value, effectiveness and barriers of NITAGs.  Method: Multi-method qualitative study comprising semi-structured interviews (IDIs) and literature review | 38 LMICs across 6 WHO regions |
| Jroundi et al [44] | Expert advisory committee | Aim: Evaluation of the Moroccan NITAG with emphasis on its functionality, quality of work processes and outputs, and its integration into the immunization policy process.  Method: Research article. Quantitative cross-sectional study | Morocco |
| Merlo et al [19] | Processes and frameworks to align economic evidence to vaccine policy priorities | Aim: Application of Accessibility and acceptability framework to identify and analyze barriers to using evidence from economic evaluation in healthcare policy  Method: Literature review | Global experience including from LMICs |
| Christen et al [11] | Use of conceptual frameworks to present vaccine impact estimates | Aim: To determine how vaccine impact estimates (VIE) are used and what actions are generated by global health organizations as a result of receiving this evidence.  Method: Qualitative research methods (FGDs) | Global experience including from LMICs |
| Green et al. [8] | Expert advisory committees | Aim: NITAG and National Certification Committee  Method: Quantitative Study | Africa |
| Steffen et al. [46] | Expert advisory committees | Aim: Describe the Role of Pasteur Institutes in vaccine decision-making  Method: Meeting Proceeding | Global experience including from LMICs |
| Sakas et al. [47] | Expert advisory committees | Aim: Assess the Role and impact of Zambia’s Inter-agency Coordinating Committee (ICC) in vaccine programming  Method: Research article; qualitative case study | Zambia |
| Uzochukwu et al. [48] | Processes and frameworks to align economic evidence to vaccine policy priorities | Aim: Health Technology Assessment (HTA) for COVID-19 vaccination  Method: Research article; mixed methods including stakeholder mapping | Nigeria |
| Molina-Aguilera et al. [45] | Expert advisory committee | Aim: Assess the perspectives on the development and use of economic evidence for immunization decision-making in a developing country  Method: A literature review of country experiences | Honduras |
| Bear et al., 2010. (47) | Processes and frameworks to align economic evidence to vaccine policy priorities | Research article;  Full Value of Vaccine Assessments (FVVA) framework to improve vaccine development, policy alignment, and decision-making​.  Method: Cross-sectional study, using accelerated failure time models to identify factors that are associated with the time to decision to adopt Hib vaccine. | LMICs |
| Hadler et al. [49] | Expert advisory committee | Aim: Evaluation of the functionality, work processes, and integration of the Moroccan NITAG into immunization policy processes​  Method: Cross-sectional study using a standardized assessment tool | Morocco |
