# Supplementary material for: Enhancing the use of economic evidence in vaccination policy and decision making in low- and middle-income countries: a scoping review of existing strategies
Source: BMJ Open. 2025 Dec 28;15(12):e103992. doi: 10.1136/bmjopen-2025-103992 (PMC12750769; doi:10.1136/bmjopen-2025-103992)
Supplement: Supplementary file 2 [file bmjopen-2025-103992.reviewer_comments.pdf]

PEER REVIEW HISTORY

BMJ Open publishes all reviews undertaken for accepted manuscripts. Reviewers are asked to complete a checklist review form and are provided with free text boxes to elaborate on their assessment. These free text comments are reproduced below.

ARTICLE DETAILS

Title (Provisional)

Enhancing the use of economic evidence in vaccination policy and decision making in low and middle-income countries: a scoping review of existing strategies

Authors

Okeke, Chinyere Cecilia; Eze, Onochie; Ekwueme, Chinazom N.; Ezechukwu, Oluchukwu; Mbachu, Chinyere O.; Uzochukwu, Benjamin; Onwujekwe, Obinna

---

VERSION 1 - REVIEW

**Reviewer**	**1**
**Name**	**García Ruiz, Antonio**
**Affiliation**	**UNIVERSITY OF MALAGA, DEP PHARMACOLOGY.**
**Date**	**06-Jul-2025**
**COI**	**None**

The paper shows high methodological coherence between the topic, the title, the problem statement, the formulation of the research problem and the objectives. All elements are aligned and contribute to a clear, relevant and well-structured research proposal in the field of public health and vaccine economics in low- and middle-income countries.

**Reviewer**	**2**
**Name**	**Ece, Berivan**
**Affiliation**	**Northwestern University, Medical Social Sceinces**
**Date**	**14-Aug-2025**
**COI**	**None**

Reviewer Comments

This manuscript presents a scoping review of strategies used to enhance the use of economic evidence in vaccination policy and decision-making in low- and middle-income countries (LMICs). The topic is timely, particularly in the context of constrained health budgets, increasing vaccine introductions, and the importance of evidence-informed decision-making. The review synthesizes data on expert advisory committees, health technology assessment (HTA), and frameworks such as the Full Value of Vaccine Assessments (FVVA), providing an organized

thematic summary. Overall, the manuscript is relevant to BMJ Open's readership, addresses an under-researched area, and has potential utility for policy-makers, researchers, and funders. However, there are methodological and reporting limitations that should be addressed to enhance reproducibility, transparency, and critical appraisal.

MAJOR ISSUES:

1. **Search strategy transparency**

- Only three databases (PubMed, HINARI, DOAJ) were searched. Authors acknowledge this limitation but they should justify the choice more explicitly and explain why other relevant databases (e.g., Web of Science, Scopus, Embase, Global Health) were not included.
- I recommend authors to explain why they used different search terms for different databases as displayed in Table 1. If that's not the case, the presented search terms are incomplete and inconsistent across databases. Researchers should be able to fully reproduce the search. For that reason, I suggest authors to consider providing full search strings for each database in an appendix.

2. **Study selection process**

- The PRISMA diagram is included, but reasons for exclusion at the full-text stage are not described. Adding these reasons in the PRISMA chart may significantly improve transparency of their scoping review.
- Eligibility criteria are not given explicitly. For example, it is unclear whether the search was supplemented with grey literature searches beyond conference proceedings. Please provide inclusion and exclusion criteria used for screening.

3. **No quality assessment**

- Authors already acknowledge this as a limitation of their study following their abstract. However, the discussion should more explicitly acknowledge how this affects interpretation of their findings.

4. **Data extraction and synthesis**

- The data extraction process could be described more systematically. For example, how were thematic categories developed? Were they pre-specified or emergent from the data?
- Tables summarizing included studies are helpful but sometimes inconsistent in the level of detail. For example,
  - In **Table 2**, Bear et al 2010, and Ducos et al (25) lack their method. Please make sure that the information presented in Table 2 is consistent and complete.
  - In **Table 2**, in the "Authors" column, some cells have numbers while others have names. They should report author names consistently. Also for the "country/region" column, specific information will be better rather than reporting as LMICs.

- Citation format in **Table 3** is not consistent. Use either numbered citations or author name consistently throughout the manuscript including tables and figures.
- Table 3: Dense content may benefit from clearer subheadings or visual separation of facilitators vs. constraints.

5. Inter-rater reliability

- Authors do not report inter-rater reliability for any stages of screening (e.g., title/abstract screening, full-text screening, data extraction). Providing inter-rater agreement metrics (e.g., Cohen's kappa, percent agreement) would strengthen the methodological rigor and enhance the transparency of their review process.

6. Interpretation and implications

- The discussion is comprehensive but could be more concise and focused on actionable implications. Currently, it repeats some results without adding interpretation.
- There is limited consideration of how context-specific factors (political stability, funding models, local research capacity) moderate the effectiveness of strategies.
- The conclusion states "low to moderate use of economic evidence" without clear criteria or data to support this categorization.

MINOR ISSUES:

Abstract:

Could be tightened; results section is long and could focus on the two main strategy categories and key findings.

Grammar and style:

Some sentences are overly long and could be restructured for clarity. For example, "Translating economic evidence generated from research into policy and practice involves an intricate process…" could be shortened without loss of meaning.

References:

- The in-text citation style is inconsistent across the manuscript, with some citations presented in numerical format and others in APA style (e.g., Lines 53 and 59). If APA style is to be used, ensure that multiple citations within parentheses are listed in alphabetical order.
- Some appear duplicated (e.g., Molina-Aguilera 2015 listed twice with slight variation). Verify formatting consistency and remove duplicates.

Formatting:

- Some minor typographical errors (e.g., stray punctuation, spacing issues, "yahooo" in author email).
- Consistency in terms: LMICS or LMICs, decision-making or decision making. Make sure you use terms consistently.
- On line 52 of the "Findings" section, I think they want to refer to Table 1 for search terms not Table 2.

Recommendation: Major revision.
The study is relevant and potentially valuable, but methodological transparency, reproducibility of the search, and critical reflection on the quality and context of included studies need strengthening before publication. Improving clarity in the results and focusing the discussion on practical implications for LMIC policy-makers will increase the paper's impact.

**Reviewer**	**3**
**Name**	**Salisbury, David**
**Affiliation**	**Royal Institute of International Affairs**
**Date**	**12-Nov-2025**
**COI**	**None**

I reviewed this article with interest as I felt that this is an important topic. Whilst there are reasonably long standing and consistent methods through which Economic Analyses have been incorporated into decision making in high income countries, the same cannot be said for processes that are used in lower income countries. Thus, there are good grounds for this review.

Because there are existing and established protocols in high income countries, as distinct from those covered in this review, I felt that it would have been helpful to provide some context of those high income countries, and how Economic Evaluation is used in immunisation decision making. The processes used in the UK and the US would be good examples where Economic Evaluation is fundamental to the recommendation process of their respective NITAGs. In the UK case, cost effectiveness that meets set criteria is a prerequisite for Government funding; and, if cost effective, the Government is obliged to provide appropriate funding.

I found there was lack of clarity about the relevance of the considerable discussions about the relevance of research to the point of the review. This is not to question the importance of research in formulating vaccine policy, but to the relevance of this issue. Much could be pruned on the discussions about research relevance. Similarly, I felt that the review went off track especially on p17 and in Table 3.

I think that it would have been helpful if the authors had included reference to the article by Shefer et al in Vaccine (2024) - 'Development of a maturity assessment tool to evaluate and strengthen National Immunization Technical Advisory Groups (NITAGs)'. Whilst this article may have come outside of the authors search criteria, its importance to the disparity of maturity of NITAGs in LMICs has relevance.

The article would benefit from edits for clarity and removal of repetitions.

VERSION 1 - AUTHOR RESPONSE

Reviewer One's comments		
The paper shows high methodological coherence between the topic, the title, the problem statement, the formulation of the research problem and the objectives. All elements are aligned and contribute to a clear, relevant and well-structured research proposal in the field of public health and vaccine economics in low- and middle-income countries.	Thank you for your kind words.	
**Reviewer Two's comments**		
**MAJOR ISSUES:**<br>**Search strategy transparency**<br>• Only three databases (PubMed, HINARI, DOAJ) were searched. Authors acknowledge this<br>limitation but they should justify the choice more explicitly and explain why other relevant databases (e.g., Web of Science, Scopus, Embase, Global Health) were not included.	These three databases were chosen following the Arksey and O'Malley framework, which calls for a repeated literature search. The literature search should map the concepts, the evidence types and the gaps in the field. The literature search does not try to be complete. PUBMED is a database that covers a range of peer-reviewed health science literature, thus a good source for studies on immunization economic evaluations and policymaking. HINARI is part of the World Health Organization's Research4Life initiative, and was selected to prioritize accessibility for researchers in LMICs, as it provides free or low-cost access to a vast array of health-related journals that may otherwise be paid for, ensuring inclusion of contextually relevant evidence from resource-constrained settings. DOAJ was included to capture high-quality open access journals, which are particularly prevalent and important in LMICs where subscription barriers limit dissemination and uptake of research. This combination balances breadth, relevance, and feasibility, especially given the review's focus on English-language publications from 2004 onward and the supplementation through reference list screening to identify additional articles. Limiting	Pg 5 lines 37 to page 6 lines 1-13.

	these databases also addressed practical constraints such as time, resources, and avoiding overlapping indexes, while still yielding a representative sample of the literature for thematic synthesis.	
I recommend that authors explain why they used different search terms for different databases,<br>as displayed in Table 1. If that's not the case, the presented search terms are incomplete and inconsistent across databases. Researchers should be able to fully reproduce the search. For<br>that reason, I suggest authors to consider providing full search strings for each database in an appendix.	Different search terms were used for different databases because the three databases have very different indexing systems, search functionalities, and content coverage. Adapting the search string to the specific features of each database is a recommended practice in systematic and scoping reviews by Cochrane and JBI guidance, to maximize the finding of as many relevant articles as possible. and avoiding excessive irrelevant results and demonstrating rigor rather than inconsistency. The authors transparently reported the exact strategy for each database in Table 1, which further strengthens the reproducibility and credibility of the review.<br><br>However, we have also provided the full search strings for each database in an appendix	Pg 6, line 29. Table 1
**Study selection process**<br>• The PRISMA diagram is included, but reasons for exclusion at the full-text stage are not described. Adding these reasons in the PRISMA chart may significantly improve<br>the transparency of their scoping review.	Thanks for the observation. We documented but did not add the reasons; it has now been inserted in the PRISMA chart.	Pg 12, Figure 1: PRISMA flow diagram
Eligibility criteria are not given explicitly. For example, it is unclear whether the search was supplemented with grey literature searches beyond conference proceedings. Please provide the inclusion and exclusion criteria used for screening.	A Table of eligibility criteria has been included. Thank you.<br><br>No separate grey-literature search was performed, as the primary aim of this scoping review was to map strategies reported in the published and programmatically cited literature rather than to achieve exhaustive capture of all unpublished material. This limitation is acknowledged in the Discussion section.	Table 2, Pgs 7 and 8
**No quality assessment**<br>The authors already acknowledge this as a limitation of their study following their abstract.	Thanks for this observation. We have included in the discussion section on limitations:	

However, the discussion should more explicitly acknowledge how this affects interpretation of their findings.	"Although a general appraisal framework was applied during data extraction and low quality or purely assertive reports contributed minimally to the final thematic synthesis. This decision aligns with common practice in scoping reviews, where the primary objective is to map the extent, range, and nature of evidence rather than to provide a definitive synthesis suitable for strong policy recommendations (Tricco et al., PRISMA-ScR, 2018; Munn et al., 2018). Nevertheless, the lack of formal quality assessment has implications for the interpretation of the findings. Themes such as the effectiveness of NITAGs, the value of capacity-building initiatives, or the reported barriers to economic evidence or uptake rely heavily on programmatic reports, expert commentaries, and observational studies that may be affected by publication bias, selective reporting, or vested interests by development partners. Consequently, while the review reliably identifies the strategies that have been attempted or advocated in LMICs, the strength of evidence supporting their effectiveness or generalizability is not conclusive. Policymakers and research funders are advised to interpret the reported successes and facilitators with caution and to prioritize strategies that have been evaluated in higher-quality comparative or longitudinal studies when such evidence becomes available. Future work in this area would benefit from systematic reviews with rigorous quality appraisal to generate more robust conclusions about which interventions most reliably enhance the use of economic evidence in immunization decision-making".	Pg 28, lines 7-26
**Data extraction and synthesis**	Thanks for the suggestion.	

• The data extraction process could be described more systematically. For example, how were thematic categories developed? Were they pre-specified or emergent from the data?	Data were analysed using thematic synthesis following the approach described by Kavanagh (2011) and adapted for scoping reviews (Brown et al., 2025). The process was iterative and combined both deductive and inductive elements:<br>Thematic development: The initial framework was pre-specified. An initial coding framework was developed deductively from the review objectives and the data-extraction template. Themes included: Types of strategies/mechanisms; Role of advisory committees (NITAGs, regional, global); Facilitators of uptake; Barriers/challenges; Reported outcomes and sustainability. The reviewers independently coded the “results/findings” and “discussion/conclusions” sections of all included studies line-by-line. New codes that did not fit the initial framework were created inductively e.g., late engagement of health economists, use of simplified one-page policy briefs, political championship, donor-driven vs. country-owned processes. There was then the development of descriptive themes, with codes grouped into descriptive sub-themes through constant comparison and regular team discussions. For example, the pre-specified theme “facilitators” expanded into the final sub-themes: capacity building/training, user-friendly evidence formats, early stakeholder engagement, local data generation, and sustainable domestic funding. Then analytical themes were generated from the descriptive themes were interpreted to generate higher-order analytical themes “institutionalization of economic evidence that requires both technical capacity and political legitimacy”.<br>Weighting of evidence and confidence assessment: During synthesis,	Pg 10, line 26 to pg 11line 8

	findings reported in multiple sources or supported by higher-quality empirical studies were given greater prominence. Single-source or purely assertive statements from low-rigor reports were retained for completeness but clearly flagged as "suggestive" in the results, e.g, Thematic data synthesis was used to collate and summarize findings from the review. e.g., NITAGs in well-documented African settings were presented with greater confidence than those from single, weak reports.	
Tables summarizing included studies are helpful but sometimes inconsistent in the level of<br>detail. For example,<br>o In **Table 2**, Bear et al 2010, and Ducos et al (25) lack their method. Please make sure that the information presented in Table 2 is consistent and complete.	The method for each of the papers has been included, thank you.	Pgs 14-17 Table 4
In **Table 2**, in the "Authors" column, some cells have numbers while others have<br>names. They should report author names consistently. Also, for the "country/region" column, specific information will be better rather than reporting as LMICs.	All author names have been included.<br><br>Some authors have the coverage as LMICs, no mention of all the countries in the methods sections, while for others, the LMICs have been written out only once and LMICs are put in brackets, as it is cumbersome and untidy to write the full list of these countries in all the cells. Thank you very much.	Pg 14, Now Table 4
Citation format in **Table 3** is not consistent. Use either numbered citations or author<br>name consistently throughout the manuscript including tables and figures.	Thank you. Numbers were used consistently, not authors' names.	Pgs 22 and 23. Table 5 now
Table 3: Dense content may benefit from clearer subheadings or visual separation of facilitators vs. constraints	This has been broken down into 7 subheadings, making it easier to understand.	Table 5, Pgs 22 and 23
**Inter-rater reliability**<br>• Authors do not report inter-rater reliability for any stages of screening (e.g., title/abstract screening, full-text screening, data extraction). Providing inter-rater agreement metrics (e.g., Cohen's kappa, percent agreement) would strengthen the methodological rigor and enhance the transparency of their review process.	Yes, this was assessed by exporting Excel with titles and abstracts from the reviewers to SPSS and calculating Cohen's Kappa.<br><br>This has been reported in the methods section: Inter-rater reliability for title/abstract screening was substantial (Cohen's κ = 0.73, 95% CI 0.69–0.77;	Pg 8, lines 36-39

	84% crude agreement) and almost perfect for full-text screening (κ = 0.89, 95% CI 0.84–0.94; 92% agreement) across the three pairs of reviewers. Emerging discrepancies were resolved by consensus.	
**Interpretation and implications**<br>• The discussion is comprehensive but could be more concise and focused on actionable implications. Currently, it repeats some results without adding interpretation.	Actionable implications have been included, and the repeated results deleted.	
There is limited consideration of how context-specific factors (political stability, funding models, local research capacity) moderate the effectiveness of strategies.	We have included a chapter on contextual issues in the discussion section:<br><br>A key insight from the reviewed evidence is that no single strategy works uniformly across LMICs; effectiveness is heavily affected by three contextual factors that future implementation efforts must assess and address. First is the political stability and governance culture. In places with politically unstable settings, even well-resourced NITAGs were sidelined or overruled. Conversely, in countries with stable multi-party systems and a tradition of technical advisory bodies e.g., South Africa, Morocco, Thailand, the same NITAG model translated into sustained influence on budget allocations. Political championship from the legislators emerged as a very strong enabler; without it, technical recommendations rarely survived budget negotiations.<br>Second is the funding and transition phase and how it is affected by Gavi. During full Gavi support, economic evidence was often produced to meet donor requirements rather than to inform local decisions, which is not an integrated process. In contrast, countries that are already self-financing, e.g., Indonesia, showed markedly higher demand from ministries of finance for budget-impact and fiscal-space analyses. Sudden	Pg 26 lines 24-p6 27 line 24

	donor withdrawal without prior local capacity building leads to the collapse of growing HTA units and a change in politically driven choices.<br>Third is the existing local research capacity. Where health institutions already had health-economics departments, e.g., South Africa and Kenya, co-production of evidence and rapid uptake were feasible. In settings with virtually no trained health economists, external consultants dominated, resulting in low ownership, limited transparency of study findings, and a lack of trust in the results. Regional initiatives such as the African Collaborating Centre for HTA only succeeded when a minimum of two to three locally trained professionals were already in place to adapt and defend the analyses. Therefore, before selecting or scaling any uptake strategy, decision-makers and technical partners should conduct a rapid contextual analysis covering these three dimensions. Strategies that ignore unfavourable political or capacity contexts are not likely to succeed. There is a need for basic domestic capacity and stable political environments before investing in advanced HTA tools or adopting sophisticated products that can support decision-making.	
The conclusion states "low to moderate use of economic evidence" without clear criteria or data to support this categorization.	This has been deleted. We now have: There is minimal use of evidence from economics in the immunization ecosystem.	Pg 2, line 36
**Abstract:**<br>Could be tightened; results section is long and could focus on the two main strategy categories and key findings.	We have revised the results section of the abstract to read this way:<br><br>Eighteen (18) eligible articles were included in the review. Across the included studies and programmatic reports, economic evidence was described as systematically requested and demonstrably influencing vaccine introduction or prioritization decisions in only 8 of 32 LMIC settings with	Pg 2. Lines 27-34

	functional NITAGs, and in fewer than 20% of documented new vaccine introduction processes since 2015. In the majority of cases, decisions were reported as being driven primarily by disease burden, political priority, donor recommendations, or historical precedent, with economic analyses either absent, produced post hoc, or acknowledged but not used as a decisive factor.	
**Grammar and style:**<br>Some sentences are overly long and could be restructured for clarity. For example, "Translating<br>economic evidence generated from research into policy and practice involves an intricate process…" could be shortened without loss of meaning.	Thanks for the observation. The long sentences have been revised.	Most pages.
**References:**<br>• The in-text citation style is inconsistent across the manuscript, with some citations presented in numerical format and others in APA style (e.g., Lines 53 and 59). If APA style is to be used, ensure that multiple citations within parentheses are listed in alphabetical order.	Thank you very much. We have done this.	Most pages
Some appear duplicated (e.g., Molina-Aguilera 2015 listed twice with slight variation).<br>Verify formatting consistency and remove duplicates.	This has been corrected and the 2nd one deleted. Thank you very much for this observation.	Pg 31, reference 32
**Formatting:**<br>• Some minor typographical errors (e.g., stray punctuation, spacing issues, "yahooo" in author email).	It has been corrected, thank you.	Most pages
Consistency in terms: LMICS or LMICs, decision-making or decision making. Make sure you use terms consistently.	Done throughout the manuscript. We are using LMICs.	Many pages
On line 52 of the "Findings" section, I think they want to refer to Table 1 for search terms not<br>Table 2.	Yes, please, we have corrected it to Table 1. Thank you.	Pg 11, line 24.
**Reviewer Three's comments**		
I reviewed this article with interest as I felt that this is an important topic. Whilst there are reasonably long standing and consistent methods through which Economic Analyses have been incorporated into decision making in high income countries, the same cannot be said	Thank you for your kind words	

for processes that are used in lower income countries. Thus, there are good grounds for this review.		
Because there are existing and established protocols in high income countries, as distinct from those covered in this review, I felt that it would have been helpful to provide some context of those high-income countries, and how Economic Evaluation is used in immunisation decision making. The processes used in the UK and the US would be good examples where Economic Evaluation is fundamental to the recommendation process of their respective NITAGs**.** In the UK case, cost effectiveness that meets set criteria is a prerequisite for Government funding; and, if cost effective, the Government is obliged to provide appropriate funding.	We have added a paragraph to the introduction section of the manuscript.<br><br>In high-income countries, the integration of economic evaluation into immunization decision-making is often formalized and follows a structure, serving as a model for global policy. For instance, in the United Kingdom, the Joint Committee on Vaccination and Immunisation (JCVI) routinely requires health economic assessments, including cost-effectiveness analyses aligned with National Institute for Health and Care Excellence (NICE) methods, to inform recommendations on vaccine inclusion in the National Health Service (NHS) schedule. The cost-effectiveness of this is usually evaluated against a threshold of £20,000–£30,000 per quality-adjusted life year (QALY), which is required for government funding. The government is obliged to provide appropriate funding if this is met. This was the case for the introduction of meningococcal B vaccine following a revised cost-utility analysis (Lieu et al,2007, Sartti et al, 2015; Sevilla 2024). Similarly, in the United States, the Advisory Committee on Immunization Practices (ACIP) considers economic evidence such as cost-effectiveness ratios and budget impact analyses, alongside clinical and epidemiological data, with explicit Center for Disease Control (CDC) guidance, ensuring transparency and methodological rigor in presentations to the committee (Pike et al, 2019; Dempsey 2008). Positive ACIP recommendations activate mandatory insurance coverage under the Affordable Care Act, expanding the role of economics in equitable access. These processes by the high-income	Pg 4, lines 13-32

	countries highlight the potential for economic evidence to drive evidence-based prioritization, yet LMICs face distinct barriers in adapting such approaches.	
I found there was lack of clarity about the relevance of the considerable discussions about the relevance of research to the point of the review.<br><br>This is not to question the importance of research in formulating vaccine policy, but to the relevance of this issue.	Although the primary focus of the review is on strategies that specifically increase the demand for and actual use of economic evidence in immunization policy and priority-setting, almost all included publications framed these strategies on "evidence-to-policy" or "research uptake" in LMICs.<br>We presented terms such as timeliness, relevance, credibility, stakeholder engagement, and policy briefs as essential enablers or constraints that determine whether economic evidence is requested, trusted, and ultimately used by NITAGs, ministries of health, and ministries of finance. This means that these are not general discussions about research uptake; they are the mechanisms through which economic evidence itself gains policy adoption in real-world LMIC immunization decisions.<br>To make it clearer and directly link these concepts to economic evidence, we have added this to the 1st paragraph of the discussion section:<br><br>The strategies identified in this review operate as institutional and procedural strategies that create formal demand for economic evidence e.g., strengthening NITAGs, legislative mandates, using decision frameworks, and knowledge-translation strategies that ensure locally produced economic analyses are perceived as timely, relevant, credible, and actionable by decision makers. Both levels are required for economic evidence to move from availability to actual influence on vaccine introduction, financing, and delivery decisions.	

Much could be pruned on the discussions about research relevance. Similarly, I felt that the review went off track especially on p17 and in Table 3.	Thank you for this helpful feedback. We agree that parts of the Discussion drifted into general evidence-to-policy that are irrelevant to the specific question of how economic evidence is made usable in LMIC immunization decisions. We also recognize that the original Table 3 ("Facilitators and constraints") reproduced broad knowledge-translation categories from the source papers instead of focusing on factors that directly affect the use of economic evidence.<br><br>We have now revised the old Table 3, which is now Table 5 following other reviewers' corrections. It focuses on factors that affect the use of economic evidence.	Table 5, Pgs 22 and 23
I think that it would have been helpful if the authors had included a reference to the article by Shefer et al in Vaccine (2024) - 'Development of a maturity assessment tool to evaluate and strengthen National Immunization Technical Advisory Groups (NITAGs)'.<br>Whilst this article may have come outside of the authors search criteria, its importance to the disparity of maturity of NITAGs in LMICs has relevance.	Thanks for suggesting this very enriching paper. We have included and used it to refine the introduction, discussion, and limitations sections of the manuscript.	
The article would benefit from edits for clarity and removal of repetitions.	This has been done. Thank you very much for this suggestion.	Many pages
